# Succinyl-proteome profiling of *Pyricularia oryzae*, a devastating phytopathogenic fungus that causes rice blast disease

**DOI:** 10.1038/s41598-018-36852-9

**Published:** 2019-03-05

**Authors:** Jiaoyu Wang, Ling Li, Rongyao Chai, Zhen Zhang, Haiping Qiu, Xueqin Mao, Zhongna Hao, Yanli Wang, Guochang Sun

**Affiliations:** 1 State key laboratory breeding base for Zhejiang sustainable pest and disease control, Institute of plant protection and microbiology, Zhejiang academy of agricultural sciences, Hangzhou, 310021 China; 2 The key laboratory for quality improvement of agricultural products of Zhejiang province, School of agricultural and food sciences, Zhejiang agriculture and forest university, Hangzhou, 311300 China

## Abstract

*Pyricularia oryzae* is the pathogen for rice blast disease, which is a devastating threat to rice production worldwide. Lysine succinylation, a newly identified post-translational modification, is associated with various cellular processes. Here, liquid chromatography tandem-mass spectrometry combined with a high-efficiency succinyl-lysine antibody was used to identify the succinylated peptides in *P. oryzae*. In total, 2109 lysine succinylation sites in 714 proteins were identified. Ten conserved succinylation sequence patterns were identified, among which, K*******K^suc^, and K**K^suc^, were two most preferred ones. The frequency of lysine succinylation sites, however, greatly varied among organisms, including plants, animals, and microbes. Interestingly, the numbers of succinylation site in each protein of *P. oryzae* were significantly greater than that of most previous published organisms. Gene ontology and KEGG analysis showed that these succinylated peptides are associated with a wide range of cellular functions, from metabolic processes to stimuli responses. Further analyses determined that lysine succinylation occurs on several key enzymes of the tricarboxylic acid cycle and glycolysis pathway, indicating that succinylation may play important roles in the regulation of basal metabolism in *P. oryzae*. Furthermore, more than 40 pathogenicity-related proteins were identified as succinylated proteins, suggesting an involvement of succinylation in pathogenicity. Our results provide the first comprehensive view of the *P. oryzae* succinylome and may aid to find potential pathogenicity-related proteins to control the rice blast disease. Significance Plant pathogens represent a great threat to world food security, and enormous reduction in the global yield of rice was caused by *P. oryzae* infection. Here, the succinylated proteins in *P. oryzae* were identified. Furthermore, comparison of succinylation sites among various species, indicating that different degrees of succinylation may be involved in the regulation of basal metabolism. This data facilitates our understanding of the metabolic pathways and proteins that are associated with pathogenicity.

## Introduction

Protein post-translational modifications (PTMs) are efficient biological mechanisms for expanding the genetic code and regulating complex cellular physiology^1,2^. In both eukaryotic and prokaryotic cells, PTMs represent an efficient strategy for increasing the functional diversity of a limited number of proteins^3^. Compared with transcription and translation, PTMs may help trigger fast responses by affecting protein localization, stability and activity levels^4^. Modifications on ε-amino groups of lysine residues have been identified in various PTMs, such as phosphorylation, acetylation, ubiquitination, methylation and succinylation^5,6^.

With the development of high-specificity antibodies and high-resolution MS techniques, more lysine modifications have been uncovered. Since the first identification in *Escherichia coli* proteins, lysine succinylation (K^suc^) has been identified in different organisms, including bacteria (*Vibrio parahemolyticus*, *Corynebacterium glutamicum* and *Mycobacterium tuberculosis*), fungi (*Saccharomyces cerevisiae* and *Trichophyton rubrum*), protozoa (*Toxoplasma gondii*), plants (*Solanum lycopersicum*, *Taxus* × *media*, *Oryza sativa*, *Brachypodium distachyon* and *Dendrobium officinale*) and mammals (*Rattus norvegicus*, *Homo sapiens* and *Mus musculus*)^7–16^. A large number of succinyl-lysine residues were identified by mass spectra (MS) and protein sequence alignments in various organisms^17^. Based on previously published data sets, proteomic lysine succinylation is evolutionarily conserved and occurs frequently in the proteins that are involved in some metabolic pathways, such as glycolysis, tricarboxylic acid (TCA) cycle and carbohydrate metabolism^10,12,18^. Thus, identifying succinylated proteins may provide useful information for biological research.

Rice (*Oryza sativa* L.) blast, a destructive disease of rice, is caused by the ascomycete *Pyricularia oryzae* (synonym, *Magnaporthe oryzae*)^19,20^. Each year, enormous reduction (approximately 10% to 30%) in the global yield of rice was caused by this disease. Due to the quickly variation velocity of *P. oryzae*, it is difficult to find rice cultivars resistant to rice blast^21^. For years, massive fungicides were used to control this disease^22^. With the increase of application dose, a series of potentially hazardous health and environmental issues have emerged^23^. Thus, it is urgent to develop an efficient and sustainable strategy to long-lasting resistance of rice to changing fungal pathogens.

Studies on *P. oryzae* pathogenic genes, which owned the potential to against fungal disease, have been carried out in recent decades. Numbers of fungal genes involved in pathogenicity have been identified in *P. oryzae*^24,25^. For example, *MPG1*, a gene expressed during infectious growth of the fungal pathogen in its host, was involved in pathogenicity from *P. oryzae*^26^. Four LIM domain proteins involved in infection-related development and pathogenicity are important regulators of infection-associated morphogenesis in the rice blast fungus^27^. Various metabolic pathways were found essential for understanding pathogenicity in *P. oryzae*. For example, the fungus can suppress host defenses via nitro oxidative stress response to facilitate the development within host cells^28^. Cellular glucose mediated TOR pathway plays an important role in the cell cycle progression during infection process of *P. oryzae*^29^. Glutaminolysis has a close relationship with appressorium formation in *P. oryzae*^30^. Lipid degradation and peroxisomal metabolism were found playing key roles in appressorial turgor generation and host invasion^31–36^. On the other hand, avirulence genes in *P. oryzae*, such as *AvrPiz-t*, *AVR-Pik*, *Avr-Pi54*, and *AVR-Pita1*, were isolated and intensively investigated for their contributions to rice resistance and interactions with resistance genes^37–41^. Additionally, host-induced gene silencing of three predicted pathogenicity genes, *ABC1*, *MAC1* and *PMK1*, significantly inhibited the development of rice blast disease^42^.

PTMs in *P. oryzae* were paid attention in very recent years. The Lysine acetylated proteins in vegetative hyphae were identified^43^ and the sirtuin mediated-deacetylation was found crucial for plant defense suppression and infection of the fungus^44^. Inhibition of histone deacetylase causes reduction of appressorium formation of *P*. *oryzae*^45^. Increasing studies indicated that lysine succinylation largely participated in the regulation of various metabolic pathways in both microbe and plant cells^9,12,13,46,47^. However, succinylated proteins in *P. oryzae* have not yet been identified so far. In the present work, we systematically identified the succinylated proteins in *P. oryzae*, which may facilitate our understanding of the metabolic pathways and proteins that are associated with pathogenicity.

## Methods

### Materials and protein extraction

The *P. oryzae* strain, Guy11 were used in our study^48^. The culture and storage of *P. oryzae* were performed using standard procedures on complete media (CM)^26^. The fungal strain was grown in CM solution, shaking at 150 rpm, in 28 °C darkness for 4 days before harvest. The samples were then placed in liquid nitrogen and sonicated three times on ice using a high intensity ultrasonic processor (type number JY92-IIN, Scientz, Ningbo, China) in lysis buffer [8 M urea, 1% Triton-100, 10 mM dithiothreitol and 0.1% Protease Inhibitor Cocktail IV, 3 μM trichostatin A, 50 mM nicotinamide, 2 mM ethylenediaminetetraacetic acid (EDTA)]. Proteins were extracted as previously described^12^. In brief, after centrifugation at 15,000 × *g* for 15 min at 4 °C, the supernatant was incubated in ice-cold acetone for more than 2 h at −20 °C. The proteins were precipitated and then redissolved in buffer (8 M urea and 100 mM NH_4_CO_3_, pH 8.0) for further tests. A 2-D Quant kit (GE Healthcare, Uppsala, Sweden) was used to determine the protein concentrations according to the manufacturer’s instructions.

### Trypsin digestion

Three protein samples were precipitated with 20% trichloroacetic acid overnight at 4 °C, and the resulting precipitate was washed three times with ice-cold acetone. Then, the protein solution was diluted in 100 mM NH_4_HCO_3_ and digested with trypsin (Promega, Beijing, China) at an enzyme/substrate ratio of 1:50 at 37 °C overnight. Then, the protein solution was reduced with 5 mM dithiothreitol at 37 °C and alkylated with 20 mM iodoacetamide for 45 min at 25 °C in the dark. To terminate the reaction, 30 mM cysteine was added and incubated for 20 min at RT. Then, to ensure complete digestion, trypsin was added at an enzyme/substrate ratio of 1:100 and incubated 4 h.

### High performance liquid chromatography (HPLC) and affinity enrichment

Three replicate samples were fractionated by high pH reverse-phase HPLC using an Agilent 300 Extend C18 column (Agilent, Beijing, China). The detailed parameters for this column are 5 μm particles, 4.6 mm ID and 250 mm length. First, the protein samples were separated using a gradient of 2% to 60% acetonitrile in 10 mM ammonium bicarbonate for 80 min at pH 10. Then, samples were combined into eight fractions for further analyses. Lysine succinylated peptides were enriched by the immune-affinity procedure. The digested samples were redissolved in NETN buffer (100 mM NaCl, 1 mM EDTA, 50 mM Tris–HCl, pH 8.0, and 0.5% Nonidet P-40) and incubated with pre-washed anti-succinyl-lysine agarose beads (PTM Biolabs, Hangzhou, China) at 4 °C overnight with gentle rotation. After incubation, the antibody beads were removed and washed carefully with NETN buffer three times, and twice with NET buffer (100 mM NaCl, 1 mM EDTA and 50 mM Tris-Cl, pH 8.0), and once with ddH_2_O. The bound peptides were eluted from the beads with 1% trifluoroacetic acid and dried in a vacuum dryer. The obtained peptides were desalted with C18 ZipTips (Millipore, Shanghai, China) according to the manufacturer’s instructions and then subjected to HPLC−MS/MS analysis.

### Liquid chromatography tandem–mass spectrometry (LC-MS/MS) analysis

The LC–MS/MS analysis was performed following the procedure described previously^12^. Briefly, the peptides were dissolved in 2% acetonitrile with formic acid and were directly loaded on an Acclaim PepMap 100 reversed-phase pre-column (Thermo scientific, Shanghai, China). An Acclaim PepMap Rapid Separation LC reversed-phase analytical column (Thermo Scientific, Shanghai, China) was used to separate the peptides. The resulting peptides were analyzed using a Q Exactive™ Plus hybrid quadrupole-Orbitrap mass spectrometer (Thermo Scientific, Shanghai, China). Then, intact peptides were detected in the Orbitrap at a resolution of 70,000 m/z 200 and were selected for MS/MS using an NCE setting of 28. Furthermore, ion fragments were detected in the Orbitrap at a resolution of 17,500. The mass spectrometry proteomics data have been deposited to the Proteome EXchange Consortium via the PRIDE partner repository with the dataset identifier PXD006778.

### Database searching

Protein and succinylation sites were identified by MaxQuant and the integrated Andromeda search engine (v. 1.4.1.2). Tandem mass spectra were compared against the several protein databases, including NCBI non-redundant (Nr) (http://www.ncbi.nlm.nih.gov/protein/), Swiss-Prot protein (http://www.uniprot.org/) and Kyoto Encyclopedia of Genes and Genomes (KEGG) (http://www.genome.jp/kegg/). Trypsin/P was determined as a cleavage enzyme, and the search has a maximum four missing cleavage sites allowance per peptide. Mass error was set to 20 and 5 ppm for the first round and main searches, respectively, and 0.02 Da for fragment ions. Succinylation on the N-terminal of a selected protein was identified as a variable modification. False discovery rate thresholds for the modification sites on peptides were specified at 1%. The parameters used in MaxQuant were set as follows: minimum length of the peptide was set at 7 and the site localization probability was set as > 0.75.

### Protein annotation and enrichment analysis

The gene ontology (GO)-based annotation of the proteome was used as the query against the UniProt-GOA database (http://www.ebi.ac.uk/GOA/). The IDs of our identified proteins were first converted to UniProt IDs. The proteins that were not annotated by the UniProt-GOA database were annotated by InterProScan software (http://www.ebi.ac.uk/interpro/) using the alignment method. The Kyoto Encyclopedia of Genes and Genomes (KEGG) pathway was annotated using the online service tool KEGG Automatic Annotation Server, and the annotation results were mapped using the online service tool KEGG Mapper. For subcellular localization predictions, the software ‘wolfpsort’ (http://psort.hgc.jp/) was used to predict subcellular localizations of identified proteins. Enrichment analyses of GO, KEGG and protein domains were performed using a two-tailed Fisher’s exact test. A correction for multiple hypothesis testing was performed using the standard false discovery rate control method. For GO and KEGG categories, *P* value < 0.05 was the threshold of significance. For the bioinformatics analyses, such as the GO-base, KEGG-base and domain-base enrichments, all of the sequences in the database were used as the background.

### Phylogenetic tree analysis

Multiple sequence alignments using the full-length protein sequences from various species were performed by ClustalW (http://www.ebi.ac.uk/Tools/msa/clustalw2/). The alignments were subsequently visualized using GeneDoc software (http://www.nrbsc.org/gfx/genedoc/), and the phylogenetic trees related to each glycolytic enzyme were constructed with 10 aligned sequences from different species using MEGA7.0 (http://www.megasoftware.net/) employing the neighbor-joining method. Bootstrap values were calculated from 1,000 iterations. The sequences of all of the proteins used in our study were obtained from the NCBI protein database.

### Motif clustering analysis

First, an enrichment analysis of all lysine succinylation substrates was carried out based on their *P* values. Based on the filter criteria, categories that were at least enriched in one cluster with *P* value < 0.05 were filtered out. Then, the *P* value matrix was calculated according to our previous work^12^. A heatmap was used to visualize the cluster membership using the ‘ggplots’ R-package (http://ggplot2.org/).

## Results

### Proteome-wide analysis of lysine succinylation sites of *P. oryzae*

**Figure 1:**
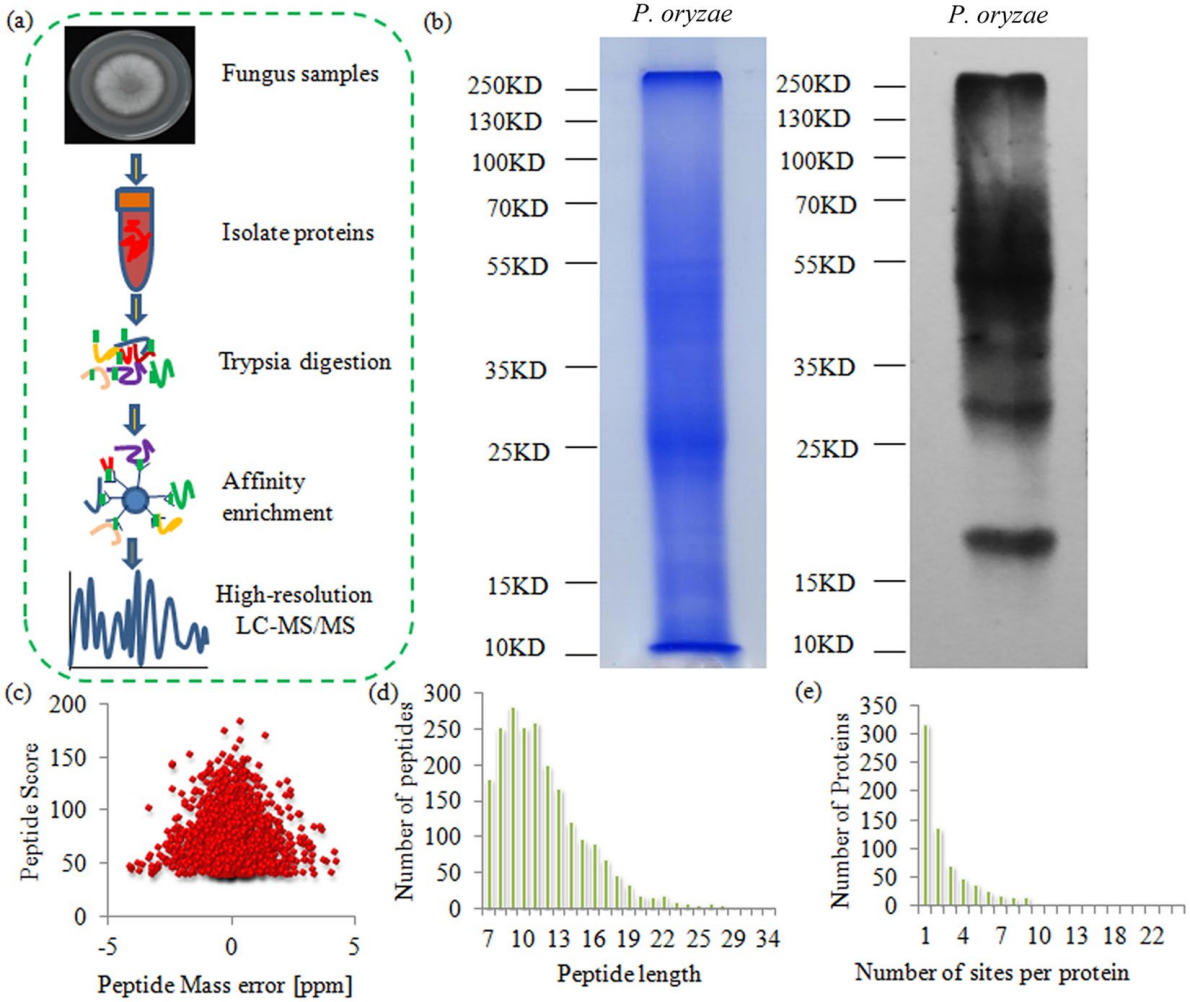
Experimental strategy and the basic information of LC-MS/MS data. (**a**) Experimental strategy for lysine succinylation identification was showed. Total proteins from *P. oryzae* were isolated and trypsin digested. All succinylated peptides were enriched by an antibody and analyzed by LC-MS/MS method. (**b**) Western-blot using a succinyl-lysine specific recognized antibody was used to check the extent of lysine succinylation on the total proteins of *P. oryzae*. (**c**) The peptides score of LC-MS/MS data. (**d**) Length distribution of succinylated peptides based on their length. (**e**) The number of proteins with different number of succinylated sites.

By combining affinity enrichment and high-resolution LC-MS/MS analysis, the systemic lysine-succinylated sites and proteins were revealed in *P. oryzae* (Fig. 1a). First, western blotting using a succinyl-lysine-specific antibody was carried out to determine the extent of lysine succinylation on the total proteins of *P. oryzae*. A wide mass-range of protein bands indicated the diversity of the succinylated proteins (Fig. 1b). Then, two quality parameters, mass error and peptide length, of the identified peptides were checked. The mass errors of the majority of succinylated peptides were lower than 0.02 Da and the lengths of most succinylated peptides varied from 7 to 18 amino-acid residues (Fig. 1c,d). The data suggested that the sample preparation and LC-MS/MS data reached approval standards.

**Figure 2:**
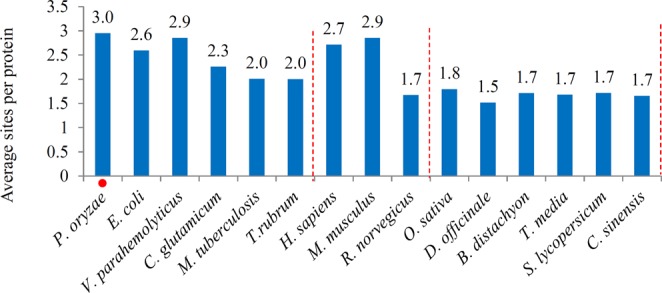
Average succinylation sites on each succinylated protein from various organisms.

Altogether, 2109 lysine succinylation sites in 714 proteins with diverse biological functions and localizations were identified (Table S1). Most of proteins contained only one to three succinylation sites (Fig. 1e). Interestingly, five proteins, including two HSP70-like protein (G4MNH8 and G4NF57), aconitate hydratase (G4N7V9), Glucose-regulated protein (G4MK90), HSP90 (G4MLM8), contained more than 20 succinylation sites, indicating a deep involvement of succinylation in various biological processes. Then, the *P. oryzae* succinylome was compared with several previously published succinylomes of other species. The average number of succinylation sites in each protein in *P. oryzae* (2.95 sites) was similar to that in *E. coli* (2.59 sites), *V. parahemolyticus* (2.85 sites), *H. sapiens* (2.72 sites) and *M. musculus* (2.85 sites), and larger than that in various plants, such as *O. sativa* (1.79 sites), *D. officinale* (1.52 sites), *B. distachyon* (1.72 sites), *T. media* (1.68 sites), and *S. lycopersicum* (1.72 sites) (Fig. 2).

### Characterization of the *P. oryzae* lysine succinylome

**Figure 3:**
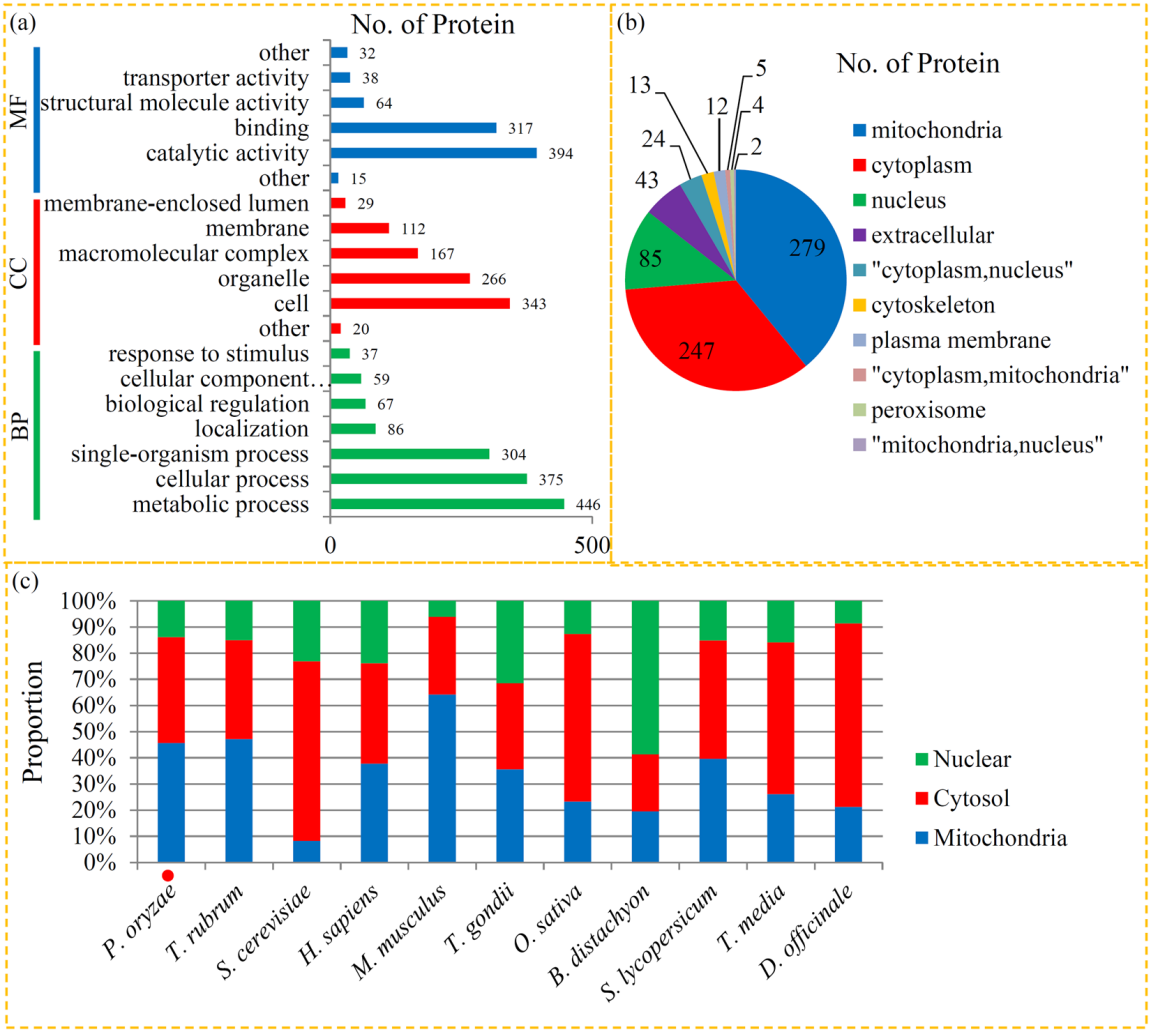
Bioinformatic analysis of lysine succinylation sites and succinylated proteins in *P. oryzae*. (**a**) GO classification for lysine succinylated proteins. (**b**) Subcellular location of lysine succinylated proteins in *P. oryzae*. (**c**) The proportion of succinylated proteins in mitochondria, cytoplasm and nucleus in various organisms.

GO functional classification is a powerful tool in understanding the possible roles of the identified succinylated proteins in various biological processes^49^. GO term classifications indicated that protein succinylation was involved in a diverse range of biological and molecular processes in *P. oryzae* (Fig. 3a). The identified results from ‘biological process’ showed that the largest number of succinylated proteins were associated with ‘organic substance metabolic process’ (level 3) and ‘organic substance metabolic process’ (level 3). In ‘molecular function’, a number of succinylated proteins were grouped into the ‘structural constituent of ribosome’ (level 3) and the ‘ligase activity’ (level 3) terms. In ‘cellular component’, most of the succinylated proteins were associated with ‘intracellular part’ and ‘cytoplasm’ (Table S2).

In *P. oryzae*, the largest group of succinylated proteins is mitochondria-located proteins (45.7%), which is the major organelle from which succinyl-CoA and succinate are mainly derived^50^. The second largest group, accounting for 40.4% of the total, is comprised of cytoplasm-located proteins, which are essential for regulating cellular metabolism. The third largest group of succinylated proteins (11.9%) were located on nucleus (Fig. 3b and Table S3). Additionally, the relative proportions of succinylated proteins in three common organelles, the mitochondria, cytoplasm and nucleus, were compared among 11 published organisms. Our data showed that *P. oryzae* possessed almost the same proportion of mitochondria-located succinylated proteins (45.7%) and cytosol-located succinylated proteins (40.4%). The relative proportions of succinylated proteins in *P. oryzae* was similar to that in *T. rubrum* and *S. lycopersicum* (Fig. 3c).

### Motif analysis in identified lysine-succinylated peptides

**Figure 4:**
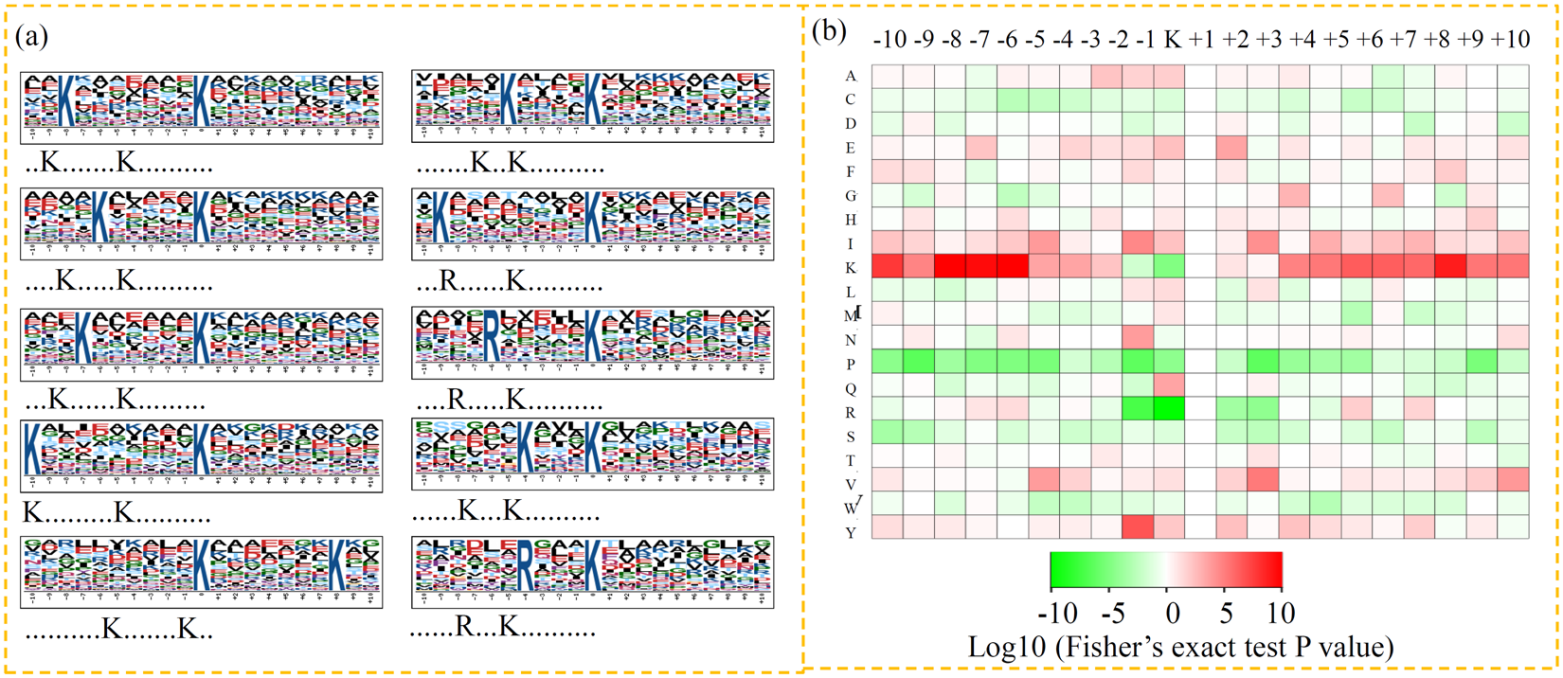
Plot shows relative abundance of amino acids flanking succinylated lysine. (**a**) The relative abundance was counted and schematically represented by an intensity map. The intensity map shows enrichment of amino acids in specific positions of succinylated lysine (10 amino acids upstream and downstream of the succinylation site). (**d**) Probability sequence motifs of succinylation sites consisting of 10 residues surrounding the targeted lysine residue using Motif-X. Seven significantly enriched succinylation site motifs were identified.

Previous studies showed that diverse amino acid patterns are present in the succinyl-peptides of different organisms^7,12,16^. In our study, the amino acid sequences surrounding succinylation sites were extracted to identify the sequence motifs in the identified succinylated proteins of *P. oryzae*. A strong bias of several flanking amino acids was uncovered upstream of the succinylated-lysine sites. To determine the general incidence of over-represented amino acids, 10 preferred sequence patterns, K*******K^suc^, K**K^suc^, K*****K^suc^, R******K^suc^, K******K^suc^, R*****K^suc^, K*********K^suc^, K***K^suc^, K^suc^******K and R***K^suc^ [*indicates a random amino acid residue and K^suc^ indicates succinylated lysine (K)], were uncovered on the basis of Motif-X with *P* < 0.000001 (Fig. 4a and Table S4). In accordance with these results, the analysis of the frequency of amino acids flanking succinylated lysine showed that the lysine (K) at −3 to −10, +4 to +10, particularly at positions −10, −6 to −8, and +8, and arginine (R) at −6 and −7, were significantly preferred (Fig. 4b).

### Enrichment analysis of the *P. oryzae* lysine succinylome

**Figure 5:**
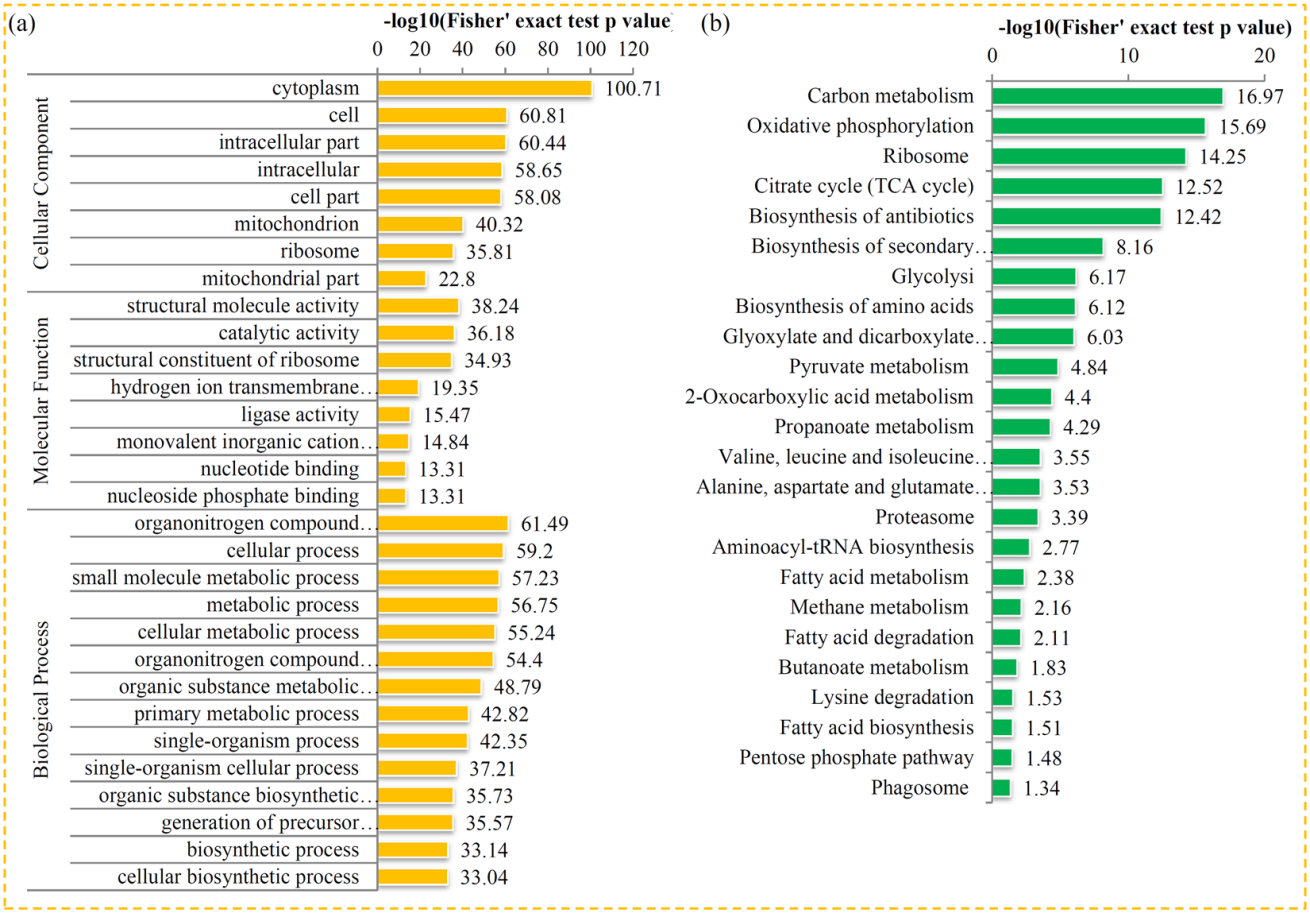
Enrichment analysis of succinylated proteins based on their annotation. (**a**) GO enrichment analysis of succinylated proteins in *P. oryzae*. (**b**) KEGG enrichment analysis of succinylated proteins in *P. oryzae*.

To investigate the preferred protein types and metabolic pathways involved in succinylation, we evaluated the GO and KEGG enrichments of the succinylated proteins in *P. oryzae*. For the GO analysis, the most significantly enriched terms in ‘biological process’ were ‘organ nitrogen compound metabolic process’, ‘cellular process’ and ‘small molecule metabolic process’. In ‘cellular component’, the succinylated proteins associated with ‘cytoplasm’, ‘cell’ and ‘intracellular part’ were the most significantly enriched. Meanwhile, in ‘molecular function’ significant enrichments of succinylated proteins were related to ‘structural molecule activity’, ‘catalytic activity’ and ‘structural constituent of ribosome’ (Fig. 5a).

For the KEGG analysis, 52 significantly enriched pathways were identified. In the succinylome of *P. oryzae*, the highest enriched metabolic pathways were ‘Carbon metabolism’ (mgr01200), ‘Oxidative phosphorylation’ (mgr00190), ‘Ribosome’ (mgr03010), ‘Citrate cycle’ (mgr00020) and ‘Biosynthesis of antibiotics’ (mgr01130) (Fig. 5b). Furthermore, the enrichment analysis of succinylated protein domains in *P. oryzae* were highly enriched in ‘NAD(P)-binding domain’, ‘Single hybrid motif’, ‘Heat shock protein 70kD, C-terminal domain’, ‘Aminoacyl-tRNA synthetase, class II’ and ‘Biotin/lipoyl attachment’ (Table S5). In summary, the enrichment analyses of GO, KEGG and protein domains suggested that lysine succinylation plays essential roles in protein biosynthesis and the central metabolism in *P. oryzae*.

### Many succinylated proteins are engaged in the several metabolic pathways

**Figure 6:**
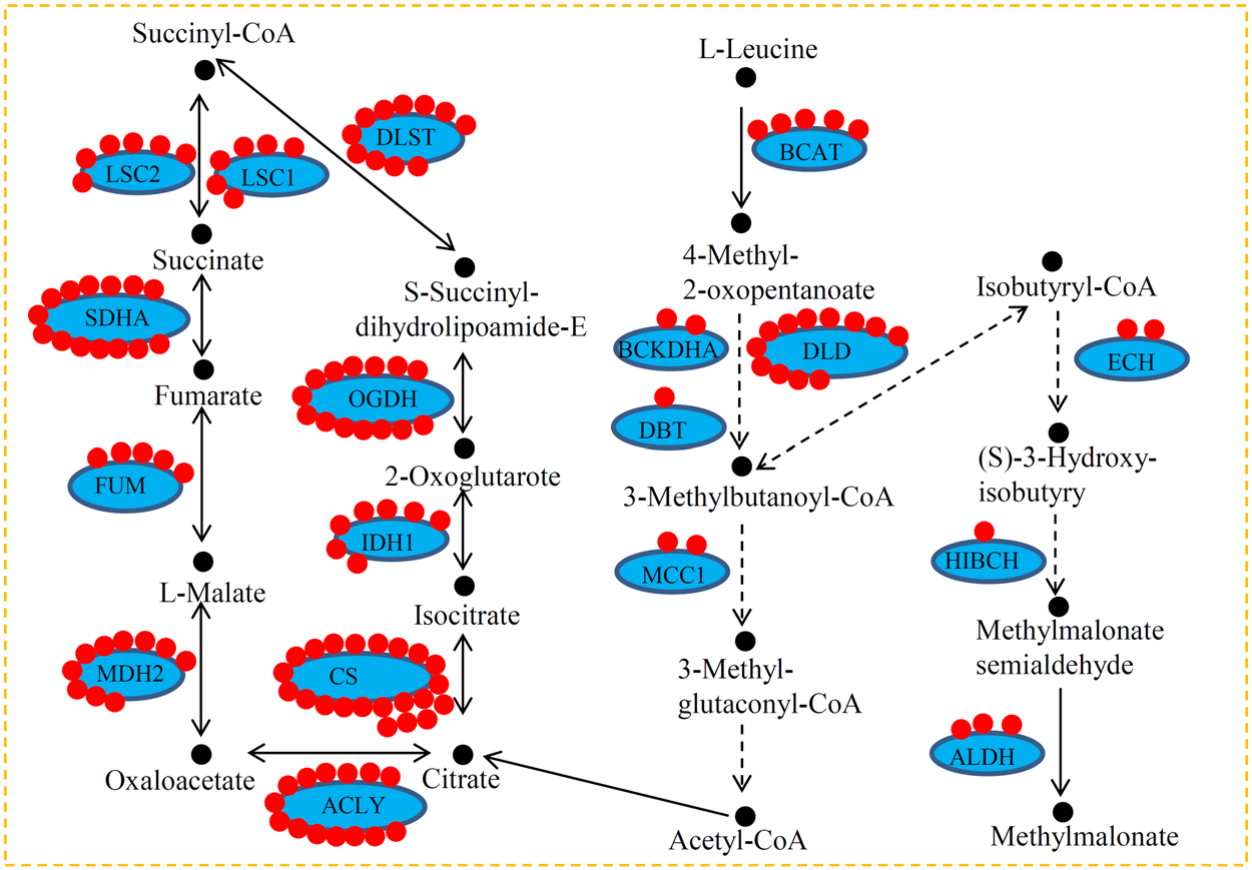
Succinylated enzymes were involved in TCA cycle and Leucine metabolism. Succinylation sites in key enzymes were involved in TAC cycle and leucine metabolism in *P. oryzae*. CS: citrate synthase; IDH1: isocitrate dehydrogenase; OGDH: 2-oxoglutarate dehydrogenase; DLST: dihydrolipoamide succinyltransferase; LSC1: succinyl-CoA synthetase 1; LSC2: succinyl-CoA synthetase 1; SDHA: succinyl-CoA synthetase; TPR:; MDH2: malate dehydrogenase 2; ACLY: ATP citrate (pro-S)-lyase; BCAT: branched-chain amino acid aminotransferase; DLD: dihydrolipoamide dehydrogenase; BCKDHA: 2-oxoisovalerate dehydrogenase E1 component alpha subunit; DBT: dihydrolipoyl transacylase; MCC1: 3-methylcrotonyl-CoA carboxylase alpha subunit; HIBCH: 3-hydroxyisobutyryl-CoA hydrolase; HIBADH: 3-hydroxyisobutyrate dehydrogenase; ATOB: acetyl-CoA C-acetyltransferase; ALDH: aldehyde dehydrogenase (NAD+).

In *P. oryzae*, 10 key TCA-related enzymes, including oxoglutarate dehydrogenase (OGDH), isocitrate dehydrogenase 1 (IDH1), citrate synthase (CS), ATP citrate (pro-S)-lyase (ACLY), malate dehydrogenase (MDH2), fumarate hydratase (FUM), succinate dehydrogenase (SDHA), succinyl-CoA synthetases 1 (LSC1), succinyl-CoA synthetases 2 (LSC2), and dihydrolipoamide succinyltransferase (DLST), were lysine-succinylated. Furthermore, eight Leucine metabolism-related enzymes, including branched-chain amino acid aminotransferase (BCAT), 2-oxoisovalerate dehydrogenase E1 component alpha (BCKDHA), dihydrolipoamide dehydrogenase (DLD), dihydrolipoyl transacylase (DBT), 3-methylcrotonyl-CoA carboxylase (MCC), enoyl-CoA hydratase (ECH), 3-hydroxyisobutyryl-CoA hydrolase (HIBCH), and aldehyde dehydrogenase (ALDH), were lysine-succinylated. Interestingly, a large number of succinylation sites were identified in OGDH (14 sites), CS (22 sites), SDHA (14 sites), ACLY (14 sites) and DLD (12 sites) (Fig. 6).

Lysine succinylation on various key enzymes in the glycolysis pathway was identified. In our study, 11 key glycolytic enzymes, including phosphoglucomutase (PGM), glucose-6-phosphate isomerase (GPI), fructose-1,6-bisphosphatase (FBP), 6-phosphofructokinase (PFK), fructose-bisphosphate aldolase (ALDO), triosephosphate isomerase (TPI), glyceraldehyde 3-phosphate dehydrogenase (GAPDH), phosphoglycerate kinase (PGK), 2,3-bisphosphoglycerate-independent phosphoglycerate mutase (GPM), enolase (ENO), and pyruvate kinase (PK), were identified as succinylated proteins. Among these proteins, PGK and GAPDH contained the largest number of succinylation sites (12 sites), while FBP and PFK contained the smallest number of succinylation sites (1 site) (Table S6).

### Identification of pathogenicity-related succinylated proteins

In the present study, 42 proteins which were previously demonstrated to be involved in pathogenicity, such as a hydrophobic-like protein (MPG1), an iscitratelyase (ICL1), a heat shock protein (SSB1) and a subtilisin-like proteinase (SPM1), were identified as succinylated proteins (Table S7). Among them, 15 proteins contained 1 succinylation site and 7 proteins contained 2 succinylation sites, while SSB1 and a 5-methyltetrahydropteroyltriglutamate-homocysteine S-methyltransferase (MGG_06712) were found containing 12 succinylation sites. The identification of these proteins suggested the association of lysine succinylation with the fungal pathogenicity.

### Comparison of succinylation sites among various species

**Figure 7:**
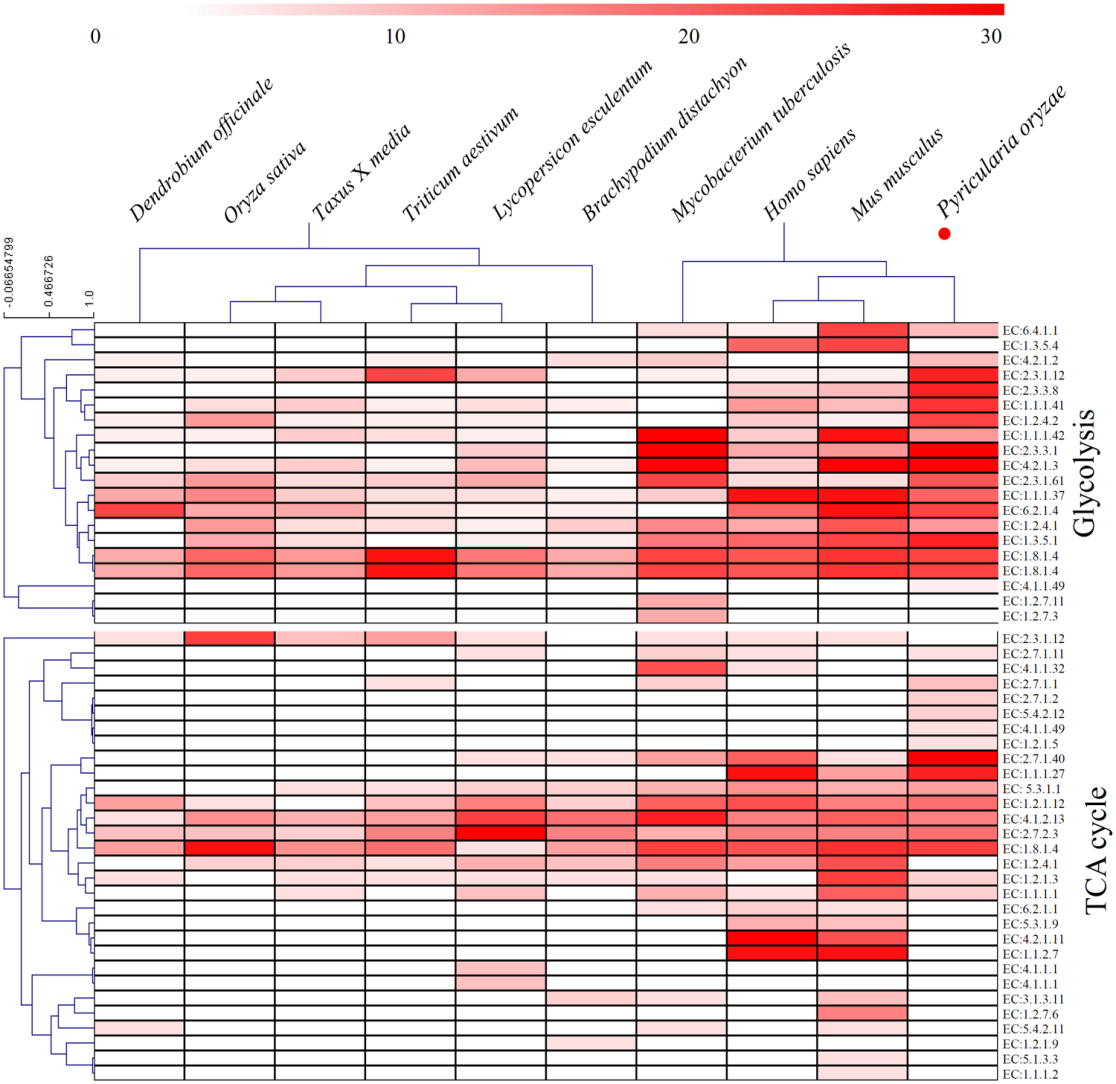
Comparison of succinylation sites in the enzymes involved in glycolysis and TCA cycle. A heatmap showed the numbers of succinylation sites in key enzymes involved in glycolysis and TCA cycle from various organisms.

Previous studies have undertaken the succinyl-proteome profiling of different species. In our study, the data extracted from two microbes (*M. tuberculosis* and *P. oryzae*), two mammals (*H. sapiens* and *M. musculus*), and six plants (*T. media*, *D. officinale*, *O. sativa*, *T. aestivum*, *L. esculentum* and *B. distachyon*) were used to evaluate the potential conserved mechanism of succinylation in the regulation of TCA cycle (Table S8) and glycolysis (Table S9). The number of succinylation sites in the 20 glycolysis-related proteins and 30 TCA-related proteins in ten representative species were counted and shown in Fig. 7. For glycolysis pathway, three enzymes, including dihydrolipoamide dehydrogenase (EC:1.8.1.4), phosphoglycerate kinase (EC:2.7.2.3) and Fructose-bisphosphate aldolase (EC:4.1.2.13), could be succinylated in all of the tested species. For TCA cycle, two enzymes, including malate dehydrogenase (EC: 1.1.1.37) and aconitate hydratase (EC:4.2.1.3), could be succinylated in all of the tested species.

## Discussion

*P. oryzae*, an ascomycete fungus responsible for the most fatal rice disease (blast), is a model species for host-pathogen interaction studies^51^. Blast disease causes enormous reduction in the yield of rice and is a major threat to food security in Asian and other regions^52^. In nature, the infection and establishment of disease depends on the asexual spores of *P. oryzae*, which colonizes leaves by producing necrotic lesions^24^. Undergoing a series of developmental and metabolic events during pathogenesis, the conidia of *P. oryzae* spread to other parts of host plants at any growth stage^20^. Further, the strains of the pathogen always show a rapid variability during rotation of rice varieties^21^. Molecular genetic analysis may help us to find potential genes to control the rice blast disease^53^. However, limited information on the PTMs has been revealed in *P. oryzae*^43–45^.

Lysine succinylation is a novel identified PTM involved in the diverse protein functions in both prokaryotic and eukaryotic cells^11^. In our study, a large number of succinylated proteins were identified in *P. oryzae* and the average succinylation sites per protein in *P. oryzae* is larger than most published species^7–15^. A higher succinylation degree of *P. oryzae* suggested an important role of succinylation in PTMs. Our data also showed that *P. oryzae* possessed the highest proportion of mitochondria-located succinylated proteins (45%), being much higher than that in and *H. sapiens* (38%). In several mammals, lysine succinylation has been identified on various mitochondrial enzymes, such as glutamate dehydrogenase, malate dehydrogenase and citrate synthase, and has positive effects on the activities of enzymes associated with mitochondrial metabolism^50,54^. The succinylation is biased to occur on mitochondrial proteins in *P. oryzae*, suggesting variability in the proportion of succinylated mitochondrial proteins^7^.

Moreover, 10 preferred sequence patterns were identified in the identified succinylated proteins of *P. oryzae*. However, some of these motifs were not unique when compared with those identified in other species, such as ‘K******K^suc^’ in *B. distachyon*, *D. officinale*, *T. aestivum*, *S. lycopersicum* and *V. parahemolyticus*^10,14,15,55^; ‘K*****K^suc^’ in *M. tuberculosis* and *B. distachyon*^15,46^; and ‘K*******K^suc^’ in *S. lycopersicum* and *V. parahemolyticus*^10,16^. This suggested that some motifs may be shared by both plants and microbes.

Ubiquitin-mediated protein degradation, a highly conserved process, occurs in proteasome and plays diverse roles in cellular processes^56^. Previous study revealed that ubiquitin-mediated proteolysis plays an important role in fungal development and pathogenicity of *P. oryzae*^57^. There was a close relationship between protein degradation and infection structure development of *P. oryzae*^58^.

Increasing evidence shows that a large portion of lysine-succinylated proteins are involved in the central metabolism, including the glycolysis pathway, TCA cycle and photosynthesis^16^. The TCA cycle and glycolysis pathway, which mainly provides the energy for life processes, interacts with sugar, fat and protein metabolism^18^. The number of succinylation sites in the key enzymes of the 10 representative species were counted and are shown in Fig. 7. For DLST, no succinylation sites were identified in the three mammals, while 10 sites in *V. parahemolyticus* and 12 sites in *M. tuberculosis* were identified^16,46^. In *P. oryzae*, 11 succinylation sites in DLST were also identified, suggesting a highly succinylated DLST in microbes. For OGDH, the greatest number of succinylation sites was identified in *P. oryzae* (14 sites) and no sites were identified in most of the tested microbes. However, for IDH1, ACLY, CS and SDHA, succinylation sites were found in all of the tested species, suggesting the succinylation of TCA-related proteins is ubiquitous during evolutionary process. The average number of succinylation sites on TCA-related enzymes among various species varied from 2.78 to 11.00. Interestingly, the average number of succinylation sites on each glycolysis-related enzyme in *P. oryzae* was larger than that in the plant species and was similar to that in mammals. Similarly, the average number of succinylation sites on each TCA cycle-related enzyme in *P. oryzae* was larger than that in plant species and was similar to that in mammals. The frequency of lysine succinylation sites, however, was varied greatly among organisms, indicating that different degrees of succinylation may be involved in the regulation of basal metabolism.

Proteinogenic amino acids are not only the building blocks of proteins, but also participate in various biological processes. Rice immune responses were reported to be induced by amino acids and their metabolites. For example, systemic disease resistance against rice blast could be induced in leaves by the treatment of rice roots with glutamate^59^. In *P. oryzae*, a number of enzymes involved in amino acid metabolism were identified as succinylated proteins, indicating an important role of succinylation in amino acid biosynthesis and metabolism of *P. oryzae*.

Notably, more than 40 well reported pathogenicity-related proteins, such as MPG1, SSB1 and 1,3-β-GTF, were identified as succinylated proteins. MPG1 is an important hydrophobic protein required for full pathogenicity of the fungus^26,60^. During the initial stages of host infection, high-level expression of the *MPG1* gene was reported to be involved in appressorium formation. Cell wall biogenesis is essential for fungal growth and pathogenesis during infection process. Cell wall biogenesis protein phosphatase SSD1 is a potential alternative regulators of cell wall biogenesis^61^. Additionally, 1,3-β-GTFs regulate the structure of the rice blast fungal cell wall during appressorium-mediated infection^62^. The presence of succinylation sites in such number of pathogenicity related proteins indicated a potential role of succinylation in the pathogenicity of *P. oryzae*. The details on the functions of these succinylation sites require further in-depth investigations.

In conclusion, we presented a comprehensive succinyl-proteome of *P. oryzae*, a filamentous heterothallic ascomycete with highly pathogenicity to rice plant. Our data are basic resources for the functional validation of succinylated proteins and a starting point for investigations into the molecular basis of lysine succinylation in *P. oryzae*.

## Supplementary information

Table S1

Table S2

Table S3

Table S4

Table S5

Table S6

Table S7

Table S8

Table S9

## References

[1] Walsh CT, Garneau-Tsodikova S, Gatto GJ (2005). Protein Posttranslational Modifications: The Chemistry of Proteome Diversifications. Angewandte Chemie International Edition.

[2] Witze ES, Old WM, Resing KA, Ahn NG (2007). Mapping protein post-translational modifications with mass spectrometry. Nature Methods.

[3] Choudhary C (2009). Lysine acetylation targets protein complexes and co-regulates major cellular functions. Science.

[4] Li H (2009). SysPTM: a systematic resource for proteomic research on post-translational modifications. Molecular & Cellular Proteomics.

[5] Yue C (2007). Lysine Propionylation and Butyrylation Are Novel Post-translational Modifications in Histones. Molecular & Cellular Proteomics.

[6] Kai Zhang YC, Zhang Z, Zhao Y (2009). Identification and Verification of Lysine Propionylation and Butyrylation in Yeast Core Histones Using PTMap Software. Journal of Proteome Research.

[7] Li X (2014). Systematic identification of the lysine succinylation in the protozoan parasite *Toxoplasma gondii*. J Proteome Res.

[8] Cheng Y, Hou T, Ping J, Chen G, Chen J (2016). Quantitative succinylome analysis in the liver of non-alcoholic fatty liver disease rat model. Proteome science.

[9] Xie L (2015). First succinyl-proteome profiling of extensively drug-resistant Mycobacterium tuberculosis revealed involvement of succinylation in cellular physiology. J Proteome Res.

[10] Jin W, Wu F (2016). Proteome-Wide Identification of Lysine Succinylation in the Proteins of Tomato (*Solanum lycopersicum*). PloS one.

[11] Weinert BT (2013). Lysine succinylation is a frequently occurring modification in prokaryotes and eukaryotes and extensively overlaps with acetylation. Cell Reports.

[12] Shen C (2016). Succinyl-proteome profiling of a high taxol containing hybrid Taxus species (Taxus x media) revealed involvement of succinylation in multiple metabolic pathways. Scientific reports.

[13] Xu X (2017). The first succinylome profile of Trichophyton rubrum reveals lysine succinylation on proteins involved in various key cellular processes. BMC genomics.

[14] Feng S (2017). Succinyl-proteome profiling of Dendrobium officinale, an important traditional Chinese orchid herb, revealed involvement of succinylation in the glycolysis pathway. BMC genomics.

[15] Zhen S (2016). First Comprehensive Proteome Analyses of Lysine Acetylation and Succinylation in Seedling Leaves of *Brachypodium distachyon L*. Scientific reports.

[16] Pan J, Chen R, Li C, Li W, Ye Z (2015). Global Analysis of Protein Lysine Succinylation Profiles and Their Overlap with Lysine Acetylation in the Marine Bacterium Vibrio parahemolyticus. J Proteome Res.

[17] Hasan MM, Yang S, Zhou Y, Mollah MN (2016). SuccinSite: a computational tool for the prediction of protein succinylation sites by exploiting the amino acid patterns and properties. Molecular bioSystems.

[18] He D (2016). Global Proteome Analyses of Lysine Acetylation and Succinylation Reveal the Widespread Involvement of both Modification in Metabolism in the Embryo of Germinating Rice Seed. J Proteome Res.

[19] Howard RJ, Valent B (1996). Breaking and entering: host penetration by the fungal rice blast pathogen Magnaporthe grisea. Annual review of microbiology.

[20] Talbot NJ (2003). On the trail of a cereal killer: Exploring the biology of Magnaporthe grisea. Annu Rev Microbiol.

[21] Ou, S. H. Variability of *Pyricularia oryzae* Cav. and its relation to varietal resistance. *Horizontal resistance to the blast disease of rice*, 49–64 (1975).

[22] Pooja K, Katoch A (2014). Past, present and future of rice blast management. Plant Science Today.

[23] Lamichhane, J. R., Dachbrodt-Saaydeh, S., Kudsk, P. & Messéan, A. Toward a reduced reliance on conventional pesticides in European agriculture. *Plant Disease* **100** (2015). 10.1094/PDIS-05-15-0574-FE 30688570

[24] Wilson RA, Talbot NJ (2009). Under pressure: investigating the biology of plant infection by *Magnaporthe oryzae*. Nat Rev Microbiol.

[25] Talbot NJ (2003). Functional genomics of plant-pathogen interactions. New Phytologist.

[26] Talbot NJ, Ebbole DJ, Hamer JE (1993). Identification and characterization of *MPG1*, a gene involved in pathogenicity from the rice blast fungus *Magnaporthe grisea*. Plant Cell.

[27] Li Y (2014). Characterisation of four LIM protein-encoding genes involved in infection-related development and pathogenicity by the rice blast fungus Magnaporthe oryzae. PloS one.

[28] Marroquin-Guzman M (2017). The Magnaporthe oryzae nitrooxidative stress response suppresses rice innate immunity during blast disease. Nature microbiology.

[29] Marroquin-Guzman M, Sun G, Wilson RA (2017). Glucose-ABL1-TOR Signaling Modulates Cell Cycle Tuning to Control Terminal Appressorial Cell Differentiation. PLoS genetics.

[30] Marroquin-Guzman M, Wilson RA (2015). GATA-Dependent Glutaminolysis Drives Appressorium Formation in Magnaporthe oryzae by Suppressing TOR Inhibition of cAMP/PKA Signaling. PLoS pathogens.

[31] Wang ZY, Soanes DM, Kershaw MJ, Talbot NJ (2007). Functional analysis of lipid metabolism in *Magnaporthe grisea* reveals a requirement for peroxisomal fatty acid beta-oxidation during appressorium-mediated plant infection. Molecular Plant-Microbe Interactions.

[32] Ramos-Pamplona M, Naqvi NI (2006). Host invasion during rice-blast disease requires carnitine-dependent transport of peroxisomal acetyl-CoA. Molecular Microbiology.

[33] Wang J (2013). PTS1 peroxisomal import pathway plays shared and distinct roles to PTS2 pathway in development and pathogenicity of Magnaporthe oryzae. PLoS One.

[34] Li L (2014). MoPex19, which is essential for maintenance of peroxisomal structure and woronin bodies, is required for metabolism and development in the rice blast fungus. PLoS One.

[35] Li, L. *et al*. Pex14/17, a filamentous fungus-specific peroxin, is required for the import of peroxisomal matrix proteins and full virulence of *Magnaporthe oryzae*. *Mol Plant Pathol* (2017). 10.1111/mpp.12487 PMC6638247 27571711

[36] Wang J (2015). One of Three Pex11 Family Members Is Required for Peroxisomal Proliferation and Full Virulence of the Rice Blast Fungus Magnaporthe oryzae. PLoS One.

[37] Devanna NB, Vijayan J, Sharma TR (2014). The blast resistance gene Pi54of cloned from Oryza officinalis interacts with Avr-Pi54 through its novel non-LRR domains. PloS one.

[38] Zhou E (2005). Evidence of the instability of a telomeric Magnaporthe grisea avirulence gene AVR-Pita in the US. Phytopathology.

[39] Li W (2009). The *Magnaporthe oryzae* avirulence gene *AvrPiz-t* encodes a predicted secreted protein that triggers the immunity in rice mediated by the blast resistance gene *Piz-t*. Mol Plant Microbe Interact.

[40] Luo CX (2005). Genetic Mapping and Chromosomal Assignment of Magnaporthe oryzae Avirulence Genes AvrPik, AvrPiz, and AvrPiz-t Controlling Cultivar Specificity on Rice. Phytopathology.

[41] Dai Y, Jia Y, Correll J, Wang X, Wang Y (2010). Diversification and evolution of the avirulence gene AVR-Pita1 in field isolates of Magnaporthe oryzae. Fungal Genet Biol.

[42] Zhu, L. *et al*. Host-Induced Gene Silencing of Rice Blast Fungus *Magnaporthe oryzae* Pathogenicity Genes Mediated by the Brome Mosaic Virus. *Genes* **8** (2017). 10.3390/genes8100241 PMC5664091 28954400

[43] Sun X (2017). Large-scale identification of lysine acetylated proteins in vegetative hyphae of the rice blast fungus. Scientific reports.

[44] Fernandez J (2014). Plant defence suppression is mediated by a fungal sirtuin during rice infection by Magnaporthe oryzae. Mol Microbiol.

[45] Izawa M (2009). Inhibition of histone deacetylase causes reduction of appressorium formation in the rice blast fungus Magnaporthe oryzae. J Gen Appl Microbiol.

[46] Yang M (2015). Succinylome analysis reveals the involvement of lysine succinylation in metabolism in pathogenic Mycobacterium tuberculosis. Molecular & Cellular Proteomics.

[47] Xu YX (2017). Quantitative Succinyl-Proteome Profiling of Camellia sinensis cv. ‘Anji Baicha’ During Periodic Albinism. Scientific reports.

[48] Leung H, Borromeo ES, Bernardo MA, Notteghem JL (1988). Genetic-analysis of virulence in the rice blast fungus magnaporthe-grisea. Phytopathology.

[49] Ashburner M (2000). Gene ontology: tool for the unification of biology. The Gene Ontology Consortium. Nat Genet.

[50] Park J (2013). SIRT5-mediated lysine desuccinylation impacts diverse metabolic pathways. Molecular cell.

[51] Dean R (2012). The Top 10 fungal pathogens in molecular plant pathology. Molecular plant pathology.

[52] Pennisi E (2010). Armed and dangerous. Science.

[53] Valent B, Chumley FG (1991). Molecular genetic analysis of the rice blast fungus, magnaporthe grisea. Annual review of phytopathology.

[54] Du J (2011). Sirt5 is a NAD-dependent protein lysine demalonylase and desuccinylase. Science.

[55] Zhang Y (2017). Global analysis of protein lysine succinylation profiles in common wheat. BMC genomics.

[56] Nath D, Shadan S (2009). The ubiquitin system. Nature.

[57] Oh Y (2012). Polyubiquitin Is Required for Growth, Development and Pathogenicity in the Rice Blast Fungus Magnaporthe oryzae. PloS one.

[58] Oh Y (2008). Transcriptome analysis reveals new insight into appressorium formation and function in the rice blast fungus Magnaporthe oryzae. Genome Biol.

[59] Kadotani N, Akagi A, Takatsuji H, Miwa T, Igarashi D (2016). Exogenous proteinogenic amino acids induce systemic resistance in rice. BMC plant biology.

[60] Soanes DM, Kershaw MJ, Cooley RN, Talbot NJ (2002). Regulation of the MPG1 hydrophobin gene in the rice blast fungus Magnaporthe grisea. Molecular plant-microbe interactions.

[61] Gerik KJ (2005). Cell wall integrity is dependent on the PKC1 signal transduction pathway in *Cryptococcus neoformans*. Molecular Microbiology.

[62] Samalova, M. *et al*. The beta-1,3-glucanosyltransferases (Gels) affect the structure of the rice blast fungal cell wall during appressorium-mediated plant infection. *Cellular microbiology* **19** (2017). 10.1111/cmi.12659 PMC5396357 27568483

